# *In vitro* antitumor activity of the ethyl acetate extract *Potentilla chinensis* in osteosarcoma cancer cells

**DOI:** 10.3892/mmr.2021.12129

**Published:** 2021-05-05

**Authors:** Guang Wan, Jin-Gang Tao, Guo-Dong Wang, Shen-Peng Liu, Hong-Xing Zhao, Qiu-Dong Liang

Mol Med Rep 14: 3634-3640, 2016; DOI: 10.3892/mmr.2016.5679

Following the publication of the above paper, a concerned reader drew to the Editor's attention that several figures bore striking similarities to other papers that were published at around the same time written by different authors based in different research institutions. Fig. 3 (in colour) was essentially the same as a greyscale figure (Fig. 4) in a paper published in *Oncology Reports*, which has now been retracted [Wan G, Tao J-G, Wang G-D, Liu S-P, Zhao H-X and Liang Q-D: 3-β-Εrythrodiol isolated from *Conyza canadensis* inhibits MKN-45 human gastric cancer cell proliferation by inducing apoptosis, cell cycle arrest, DNA fragmentation, ROS generation and reduces tumor weight and volume in mouse xenograft mode. Oncol Rep 35: 2328-2338, 2016]. Furthermore, Figs. 5 and 6 in the above paper appeared to share data with Figs. 7 and 11, respectively, in a paper published in *Phytomedicine* [Sui C-G, Meng F-D and Jiang Y-h: Antiproliferative activity of rosamultic acid is associated with induction of apoptosis, cell cycle arrest, inhibition of cell migration and caspase activation in human gastric cancer (SGC-7901) cells. Phyomedicine 22: 796-806, 2015].

After having conducted an independent investigation in the Editorial Office, the Editor of *Molecular Medicine Reports* has determined that the above paper should be retracted from the Journal on account of a lack of confidence concerning the originality and the authenticity of the data. The authors were asked for an explanation to account for these concerns, but the Editorial Office never received any reply. The Editor regrets any inconvenience that has been caused to the readership of the Journal.

